# Toward Sub-Diffraction Imaging of Single-DNA Molecule Sensors Based on Stochastic Switching Localization Microscopy

**DOI:** 10.3390/s20226667

**Published:** 2020-11-21

**Authors:** Seungah Lee, Indra Batjikh, Seong Ho Kang

**Affiliations:** Department of Applied Chemistry and Institute of Natural Sciences, Kyung Hee University, Yongin-si, Gyeonggi-do 17104, Korea; moon1131@gmail.com (S.L.); indak09@gmail.com (I.B.)

**Keywords:** single-DNA molecule sensor, DNA nanoarchitectures, direct stochastic optical reconstruction microscopy, sub-diffraction imaging, super-resolution microscopy

## Abstract

The natural characteristics of deoxyribonucleic acid (DNA) enable its advanced applications in nanotechnology as a special tool that can be detected by high-resolution imaging with precise localization. Super-resolution (SR) microscopy enables the examination of nanoscale molecules beyond the diffraction limit. With the development of SR microscopy methods, DNA nanostructures can now be optically assessed. Using the specific binding of fluorophores with their target molecules, advanced single-molecule localization microscopy (SMLM) has been expanded into different fields, allowing wide-range detection at the single-molecule level. This review discusses the recent progress in the SR imaging of DNA nano-objects using SMLM techniques, such as direct stochastic optical reconstruction microscopy, binding-activated localization microscopy, and point accumulation for imaging nanoscale topography. Furthermore, we discuss their advantages and limitations, present applications, and future perspectives.

## 1. Introduction

One of the basic molecules in the central dogma of molecular biology is deoxyribonucleic acid (DNA), which is a fundamental instrument for biological inheritance and genetic information coding. DNA has a uniquely sequenced structure and molecular recognition properties, which enable its application as a tool and target in nanotechnology [1]. Two or three-dimensional (2D or 3D) DNA structures have been reported with different sizes, shapes, and functions for specific applications. DNA is a fundamental building block in complex nanoarchitectures with well-characterized features, including a 3.4 nm helical repeat and 2 nm diameter [2]. Assemblies constructed with oligonucleotides under certain physical conditions can be employed to explore the interactions among target molecules (such as proteins) with nanoscale precision through the hybridization of complementary staple (i.e., short DNA oligomers with 20–60 nucleotides) strands. Programmable and self-assembling DNA nano-objects, including ribbons [3], tiles [4], lattices [5], and bricks [6], can be assembled with various other nanomaterials. One of the functional tools created using this principle, first reported by Fu and Seeman, is DNA origami, which comprises a single long strand of DNA that self-assembles with short staple strands in particular regions [7]. The attachment points of two neighboring double helices form a DNA crossover, which allows the control of the shape of the DNA nanostructure by the selection of complementary segments [8]. The unique positioning of the staple strand in DNA origami has attracted considerable attention in biological studies, initially as a target for label-free ribonucleic acid (RNA) [9]. In addition, the DNA tile concept is based on combinations of DNA-branched junctions with sticky-end associations that form a 2D lattice. Moreover, DNA sensors have been developed for single-nucleotide polymorphism detection, enabling single-molecule visualization [10]. DNA bricks have been developed through nanofabrication techniques based on synthetic short DNA strands that are capable of self-assembly [6]. Many other applications have also been reported, such as molecular tracking using DNA walkers [11] or DNAzymes [12]. In addition, the staple joints of DNA origami rulers have been used for calibration in super-resolution (SR) microscopy [13].

To observe the functionalized nanoscale assemblies of DNA, single-molecule detection methods have been established based on numerous technologies, including SR microscopy. The breakthrough of SR microscopy has enabled the detailed visualization of various nanostructures. In particular, the ability to locate each fluorophore with precision using single-molecule localization microscopy (SMLM) has expanded the application of SR microscopy. SMLM methods use sequence-guided DNA molecules owing to their unique sequence and hybridization, which is more suitable for single-molecule imaging. At present, stochastic switching and readout approaches, such as photoactivated localization microscopy (PALM) [14] and stochastic optical reconstruction microscopy (STORM) [15], are employed by researchers in SR microscopy. The fluorescent dyes in stochastic SR microscopy are “switched” to localize DNA molecules through the SR imaging of a nonfluorescent dark state and fluorescent bright state, with a sub-diffraction resolution. Furthermore, direct STORM (dSTORM) has been developed using organic fluorophores that operate as reversible photoswitches in reducing and oxidizing buffering systems [16]. Another form of stochastic switching microscopy, fluorescence PALM (fPALM), uses fluorescent proteins that stochastically switch, enabling single-molecule SR imaging. Furthermore, DNA-based point accumulation for imaging in nanoscale topography (DNA-PAINT) [17] is an approach that exploits the apparent “blinking” of a target based on the transient binding of a dye-labeled oligonucleotide (imager strand) to a target-binding complement (docking strand).

This review examines the sub-diffraction imaging of DNA molecular sensors based on stochastic SR microscopy techniques. This review is organized based on the determination methods used. In Section 2, we first highlight the detection and application of DNA nanostructures in stochastic switching-based localization microscopy, i.e., STORM; binding-activated localization microscopy (BALM), including DNA-binding dyes with adjustable chemical conditions; and DNA-PAINT, which uses DNA in different approaches for detection applications. Section 3 concludes the discussion and offers future perspectives for DNA molecule SR imaging and the applications of DNA nanoarchitectures in SMLM.

## 2. Single-DNA Molecules as Targets in Stochastic Switching-Based Localization Microscopy

**Figure 1:**
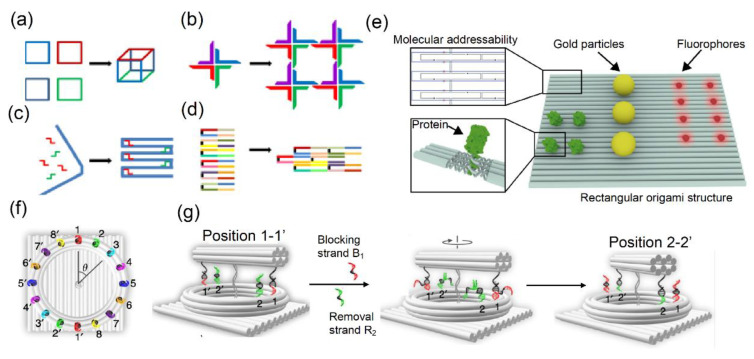
Different concepts of DNA assembly: (**a**) a finite-sized nanostructure; (**b**) DNA tiles assembled as a 4 × 4 2D square lattice, (**c**) scaffold build based on long (blue) and short (red and green) DNA oligonucleotides, (**d**) DNA bricks constructed by short oligonucleotides, and (**e**) DNA nanostructures comprising different molecules such as protein (green), gold particles (yellow), or fluorophores (red). Top left: Detailed illustration of staple and scaffold strands. Bottom left: Specific sequence-expanded staple strands. (**f**) Foothold arrangement around the ring tracks eight different colors of stepwise plasmonic nanoclock rotation steps corresponding to *Δθ* = π/8. (**g**) The principle is based on the release and capture mechanism powered by the interaction between DNAzyme and RNA in a stepwise rotation with additional blocking strands and removal strands by toehold-mediated strand displacement reactions. Images (**a**–**d**) were reproduced with permission from Gradišar et al., published by BioMed Central, 2014 [18], (**e**) was reproduced with permission from Schlichthaerle et al., published by Elsevier, 2016 [23], and (**f**,**g**) were reproduced with permission from Xin et al., published by Nature Publishing Group, 2019 [22].

DNA-powered imaging technologies are ideal for investigating the interactions among molecules and have been developed and optimized as highly effective visualization methods in biology. DNA nanoarchitectures vary in size and structure based on their assembly: from several average-sized DNA oligonucleotides to 2D and 3D spaced DNA assemblies filled with short DNA bricks comprising long and short nucleotide strands (Figure 1a–d) [18]. The initial approach for the construction of nanostructured DNA was based on a Holliday junction with four double helices at the center point of four DNA strands, as first reported by Robin Holliday in 1964 [19]. Following the development of this DNA nanostructure, DNA tiles were developed based on a four-way junction with sticky ends for hybridization, leading to stable higher-order structures [20]. Another approach for the construction of DNA lattice nanostructures involves self-assembly through blunt-ended interactions and sticky-ended hybridization [21]. One of the building blocks of these 2D and 3D nanoarchitectures is DNA origami scaffolds with long strands conjugated with short staple strands. Recently, Xin et al. applied toehold-mediated strand displacement nanotechnology to develop a plasmonic nanoclock based on functional nucleic acid, i.e., DNAzyme and RNA interactions, which enabled the rotation of a 10 helix DNA origami assembly (Figure 1f, g) [22]. One advantage of using DNA to assemble 2D and 3D nanoarchitectures is the ability to interface and label any position with other functional molecules. Metallic nanoparticles, proteins, fluorophores, dye molecules, quantum dots, and other organic compounds (Figure 1e) [23] have been used as bioimaging probes to investigate biological mechanisms such as cell signaling pathway.

### 2.1. dSTORM

**Figure 2:**
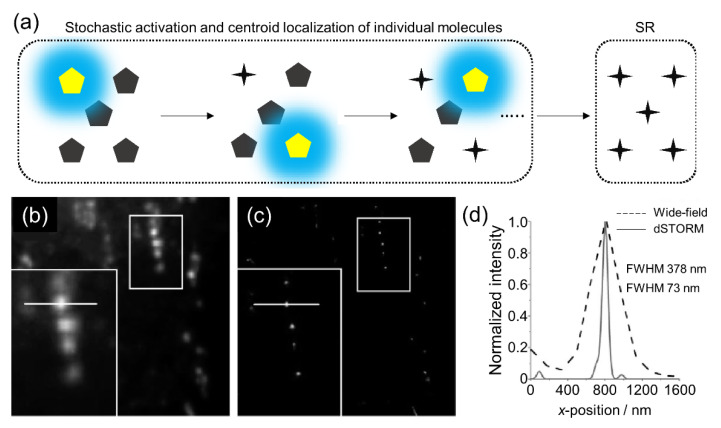
(**a**) Schematic showing the repeated cycling principles of STORM. Comparison between the (**b**) wide-field and (**c**) dSTORM images of mitochondrial DNA with (**d**) line-intensity profiles. Images (**b**–**d**) were reproduced with permission from Benke et al., published by John Wiley and Sons, 2011 [25].

In 2006, Rust et al. invented a single-molecule localization method named STORM, which is based on the “bright” and “dark” state transition of a fluorophore [15]. The principle of STORM involves super-localization by the photon emission of optically photo-switchable molecules in the transition between the “bright” and “dark” state or photobleaching (Figure 2). The localization of many switching fluorophores by controlling on and off switches within the diffraction-limited spatial resolution is the main advantage of STORM. Later, dSTORM was introduced by Heilemann et al. as a variation of STORM [16]. This technique uses conventional cyanine dye-labeled dsDNA analyzed using a Gaussian function with the determination of the precise position as a reversible cycle between a “bright” and a “dark” state using photophysical and photochemical transitions without requiring an activator fluorophore. dSTORM has a simple and inexpensive setup and uses small fluorophores exhibiting extended and reasonable excitation stability; however, there are some limitations in imaging quality [24].

**Figure 3:**
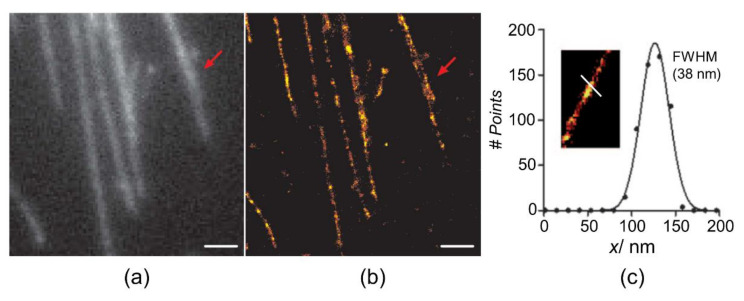
(**a**) Wide-field image of λ-DNA labeled with YOYO-1 dye, observed via SR imaging with an integration time of 100 ms and (**b**) reconstructed image with the density of localization. (**c**) The resolution across the DNA is 40 nm with Gaussian fitting of the line. The region indicated by red arrows shows the difference in the observation between the wide-field and SR images of dsDNA (scale bar: 1 µm). Reproduced with permission from Flors et al., published by Wiley-VCH Verlag Gmbh, 2018 [26].

Steinhauer et al. measured the distance of single molecules by binding fluorescent dyes to the diagonal ends using rectangular DNA origamis (100 nm × 70 nm) that had a diagonal distance within the diffraction limit (89.5 nm). Such DNA origami molecular rulers can be used as quantification standards for SR microscopes [13]. Flors et al. showed that commercially available intercalating cyanine dyes can be used to easily label and image DNA using a single laser to achieve SR (Figure 3a,b) [26]. Accordingly, they studied the properties of photo-switching and photo-blinking properties of DNA-intercalating cyanine dyes with monomeric and dimeric structures under different buffer conditions. Their results showed that these photophysical characteristics could be manipulated in the SR imaging of DNA [27]. However, they stated that DNA staining with a YOYO-1 dye could be achieved with reconstruction in SR with precise resolutions under 40 nm (Figure 3c) [26].

**Figure 4:**
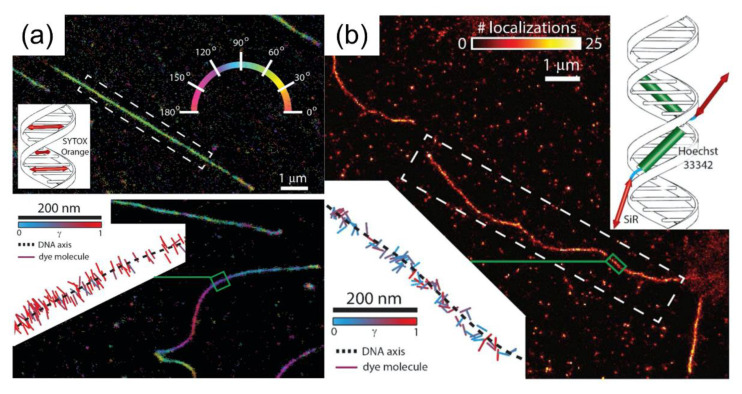
Super-resolved images and single-molecule orientation measurements acquired using DNA-staining dyes: (**a**) SYTOX Orange and (**b**) SiR-Hoechst. The images were reproduced with permission from Backer et al., published by the Optical Society of America, 2016 [28].

The assessment of intercalating dyes as a sensor for conformational changes induced by DNA-binding proteins at the single-molecule level was performed by Backer et al. Their approach, which was based on molecular localization, demonstrated super-resolved imaging through binding-induced switching “on” of the fluorescent dye. The use of polarization-dependent localization microscopy allowed access to the dynamics of single-molecule rotation and enabled the observation of distortion of a single-DNA molecule when characterization was performed using various DNA-staining dyes. From the localization measurements, it was determined that the SYTOX Orange (intercalating dye to DNA molecule) binds between adjacent DNA base pairs (Figure 4a, inset), under the application of absorption dipole moments in a perpendicular position. Owing to the indirect binding of SiR-Hoechst (minor groove binder to DNA molecule), the absorption dipole moments of silicon–rhodamine were not constrained (Figure 4b, inset) [28].

**Figure 5:**
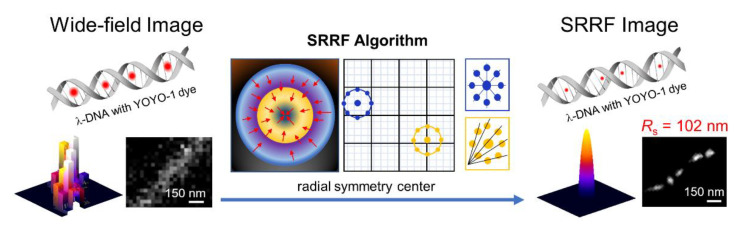
Schematic illustration of single-DNA molecule detection using the TIRF-based SRRF stream module. The image was reproduced with permission from Nguyen et al., published by Wiley-VCH Verlag Gmbh, 2020 [30].

Moreover, the advanced imaging system based on combination with other detection techniques, such as total internal reflection fluorescence (TIRF) or highly inclined laminated optical sheet (HILO), gives more accuracy over the diffraction limit than conventional STORM. Gustafsson et al. suggested an SR radial fluctuation (SRRF) algorithm as a novel analytical approach [29]. This is a fast, thresholdless algorithm that involves the temporal analysis of subpixel geometric measurements applied to an image sequence. Nguyen and Kang compared TIRF-based SRRF and dSTORM using a YOYO-1 intercalating dye with a single *λ*-DNA [30]. The distance between fluorophores in the SRRF images was 102 nm with a 1:300 ratio (dye:DNA), which was similar to that in the images detected by dSTORM with the same ratio. However, if the dye:DNA ratio decreases (1:50), SRRF cannot distinguish single fluorophore signal in DNA molecule, whereas dSTORM can successfully obtain an image with a 17 nm distance between localized fluorophores. TIRF-based SRRF imaging can perform fast image processing; thus, it is a novel, time-efficient analytical module with simple operation (Figure 5).

### 2.2. BALM

**Figure 6:**
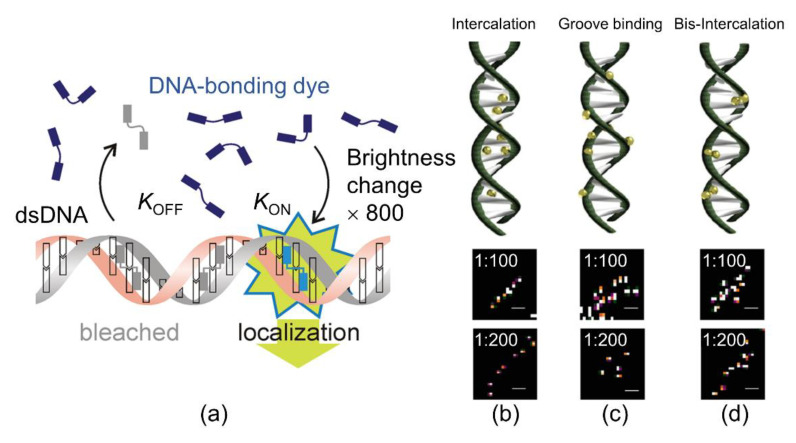
(**a**) Principle of binding-activated localization microscopy (BALM). When the target molecules bind to the free dyes, they turn bright and can be localized until they bleach or separate from the target. *k*_off_ defines the DNA unbinding rate, and *k*_on_ defines the DNA-binding kinetics and concentration of the dye in solution. The diagram was reproduced with permission from Schoen et al., published by American Chemical Society, 2011 [31]. Various binding approaches of YOYO-1 in the DNA molecule including (**b**) intercalation, (**c**) groove binding, and (**d**) bis-intercalation. (Top) Binding types of YOYO-1 with λ-DNA based on the position in the strands. (Bottom) Specific regions of YOYO-1 in λ-DNA for the 1:100 and 1:200 (dye:DNA bp) ratios, representing each binding mode (Top). The image was reproduced with permission from Park et al., published by Elsevier Science Inc., 2018 [32].

As an alternative strategy to photo-switching, Schoen proposed BALM, which uses fluorophores that are “switched on” after binding to the target structure, directly exploiting this property to localize them under dynamic binding conditions (Figure 6a) [31]. Generally, BALM can visualize biological or synthetic structures with nanoscale resolutions using target-binding dyes. It uses DNA-binding dyes that show a strong fluorescence enhancement once bound to the dsDNA. The BALM technique was applied by Park et al. to single-DNA molecules to detect the SR morphological distribution of the YOYO-1 dye. Owing to the various binding approaches of YOYO-1, the dye was disseminated homogenously and non-homogenously in the DNA molecule (Figure 6b–d). In their results, BALM images were used for the determination of YOYO-1 to localize the center point of the DNA molecule, which shows that YOYO-1 was intercalated between base pairs (Figure 6b). Conversely, YOYO-1 was observed in groove binding (Figure 6c) and the middle of the planar bases of DNA, suggesting the bis-intercalation (Figure 6d) mode [32].

**Figure 7:**
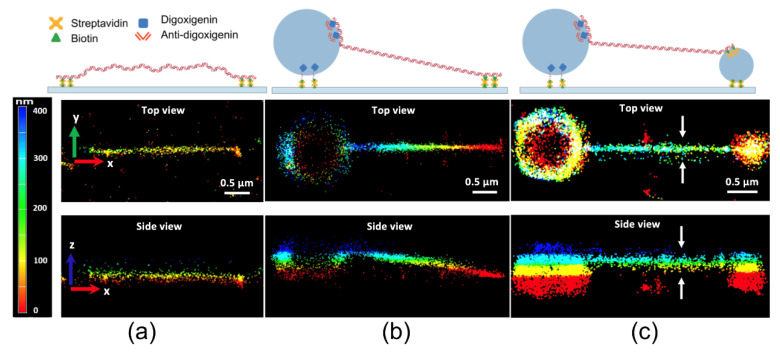
(**a**) Three-dimensional BALM images of 10 kb linear dsDNA tethered to the surface of the glass at both ends, (**b**) tethered to the surface at one end and to a 1 μm diameter bead at the other end, and (**c**) tethered to a 1 μm diameter bead at one end and a 0.4 μm diameter bead at the other end. The image was reproduced with permission from Yardimci et al., published by Nature Publishing Group, 2020 [33].

Yardimci et al. accomplished BALM with a combination of fluorescent double-stranded DNA intercalators and optical astigmatism to acquire 3D SR images of DNA, using microspheres to make DNA visible in the 3D SR images (Figure 7) [33]. DNA with a biotin-modified end is tethered to a streptavidin-coated glass coverslip (Figure 7a). The color-coded images of DNA molecules demonstrate that the axial position of the DNA increased from the surface-tethered end toward the microsphere-tethered end (Figure 7b). The 3D SR images of the double-microsphere-tethered DNA show the stretching of the entire molecule above the surface (Figure 7c).

Recently, Meijering et al. explained that diffraction-limited inverse imaging can reveal molecular mobility at a temporal resolution of approximately 0.2 s, and the method works both with DNA-intercalating and non-intercalating dyes using inverse BALM. The main concept relies on the label-free imaging method in the absence of a fluorescence signal, where the protein patches bind to the DNA [34].

### 2.3. DNA-PAINT

Sharonov and Hochstrasser made the first attempt at conducting PAINT based on the blinking of a diffusing probe, Nile red, with large unilamellar vesicles based on their well-characterized collision kinetics [35]. Nile red is a lipophilic dye that targets a lipid core and exhibits fluorescence in a hydrophobic environment. Interestingly, they found one or two instances of fluorescence that localized the single vesicles to characterize single-molecule imaging. Approximately 10% of the signal was observed using individual vesicles in 20 ms frames. They explained the temporary binding of Nile red by discontinuous collisions that target large unilamellar vesicles, which led to the invention of “point accumulation for imaging in nanoscale topography” (hereinafter, PAINT technique). This technique was improved to capture a high-resolution image to overcome the unclear emission caused by the diffraction limit. Besides using the interaction between dye and target, PAINT is an advanced and efficient imaging technique.

**Figure 8:**
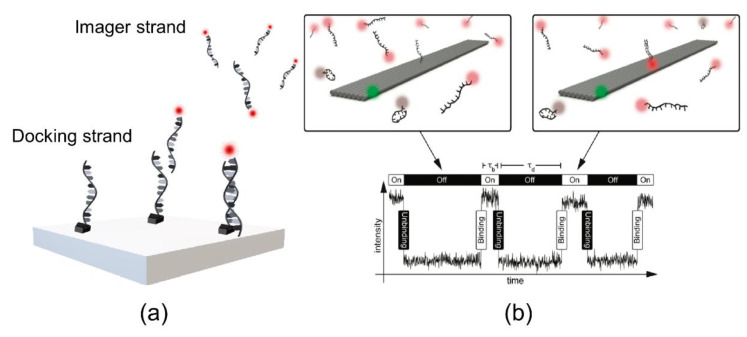
DNA-PAINT (point accumulation for imaging in nanoscale topography). (**a**) Imager strand labeled with fluorescence dye and the docking strand on the surface of a synthetic DNA origami. (**b**) The transient binding (hybridization) of the imager strand to the docking strand will switch the fluorescence “on” and “off.” The image was reproduced with permission from Jungmann et al., published by American Chemical Society, 2010 [17].

As previously mentioned, Jungmann et al. employed PAINT for the visualization of DNA using the assembly of a nanoscale molecule and a synthetic DNA scaffold, termed as DNA origami [17]. In this work, rectangular origamis (approximately 20 nm × 260 nm) were used as three docking sites (spaced at 130 nm) to bind fluorophore-labeled DNA imager strands (Figure 8). The main principle of DNA-PAINT relies on imager strands (a 7–11 nucleotide DNA fragment) labeled with a fluorophore that binds to a complementary docking strand [36]. The transient binding of the docking strands and the imager strands enables detection of sub-diffraction imaging. Furthermore, DNA-PAINT imaging is dependent on the position of the docking strand on the DNA origami. The unique feature of DNA-PAINT is transient binding, which overcomes photobleaching with dye-labeled imager strands. PAINT has advantages over other methods because it solves the problem of photobleaching. The transient binding of an imager strand to a docking site allows the continuous replacement of photobleached probes with new ones, rendering PAINT free from photobleaching. Moreover, further developments in DNA-PAINT research have been achieved by improving performance through increased imaging speed [37] and expanding its field of application, among others.

In 2014, Raab et al. used DNA origami nanorulers to overcome the discrepancy of localizing single objects and separating two objects by resolving two docking sites at distances of 18, 12, and 6 nm, using SR techniques, mainly DNA-PAINT [38]. In 2017, they proposed an assay based on a rectangular DNA origami with two different structures to distinguish the separation of emitters and a gold nanoparticle. In a symmetrical triangle (50 nm in length), the DNA marks were arranged into one structure. Thus, the localization of sample structures can be calculated based on the determination of three different structures [39]. Recently, Stehr et al. studied DNA-PAINT imaging using TIRF-based flat-top beam illumination [40]. The main concept was to capture the blinking events missed in the DNA-PAINT by converting Gaussian excitation into a flat-top profile. Compared with a Gaussian illumination, a flat-top laser beam can increase the blinking event of the imager strand bound to the docking site, thereby providing a more accurate image.

**Figure 9:**
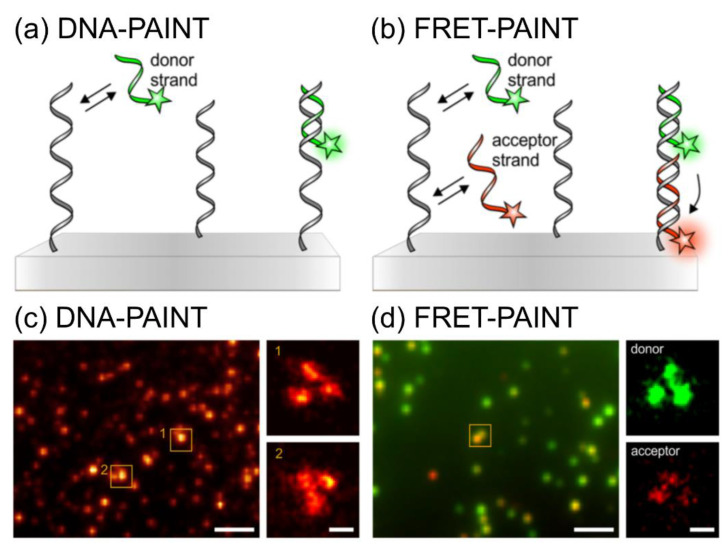
Principle of DNA-PAINT and FRET-PAINT. (**a**) Schematic illustration of DNA-PAINT based on a DNA origami with a donor strand (green highlighted strand) and a docking site (gray strand). (**b**) Illustration of FRET-PAINT with an additional acceptor strand (red highlighted strand). (**c**) DNA-PAINT-based DNA origami image labeled with docking sites (left, scale bar: 2 µm) with regions of interest of single-DNA origami (right, scale bar: 50 nm), observed at 15,000 frames with the calculation of standard deviation. (**d**) Permanent complementary binding of the donor (green, nine nucleotides) and acceptor (red, 10 nucleotides) strands with the docking site of DNA origami images was obtained by FRET-PAINT (left, scale bar: 2 µm). Because of the excitation of the donor strand, the acceptor emission gives FRET-PAINT signal of the binding of both strands with their target sites. SR images are created with the localization of single dyes (right, scale bar: 50 nm). The images were reproduced with permission from Deußner-Helfmann et al., published by American Chemical Society, 2018 [42].

In terms of background-noise-free imaging, DNA-PAINT uses Förster (fluorescence) resonance energy-transfer (FRET)-based probes to overcome the background signals of diffused imager strands and facilitate fast imaging [41]. Figure 9 shows the principles of DNA-PAINT and FRET-PAINT [42]. In DNA-PAINT, a short, fluorophore-labeled DNA strand (imager strand) transiently binds to a complementary target sequence (docking strand) and generates a detectable signal (Figure 9a). In FRET-PAINT, two imager strands simultaneously bind to one target sequence: the first is a donor strand, and the second contains an acceptor fluorophore. The binding of both strands affords a detectable FRET signal in the acceptor fluorophore upon excitation by the donor (Figure 9b). The specific binding of the docking site and imager strand in DNA-PAINT has certain disadvantages: minor non-specific binding occurs under hydrophobic conditions; the requirement of high concentrations of the imager strand to achieve high-resolution images; and high background noise with high concentrations of dye-labeled imager strands (500 pM–10 nM concentration). The application of FRET-based probes for DNA-PAINT overcomes these disadvantages for background-noise-free, fast, and accurate binding [43].

By combining FRET-PAINT and real-time confocal microscopy, the 3D structure of thick tissue samples can be reconstructed with high speed (approximately 30 times faster than the original speed) and with less background noise. FRET-based DNA-PAINT imaging with TIRF allows the acquisition of high-resolution images, even with a high concentration of the imager strand [43].

**Figure 10:**
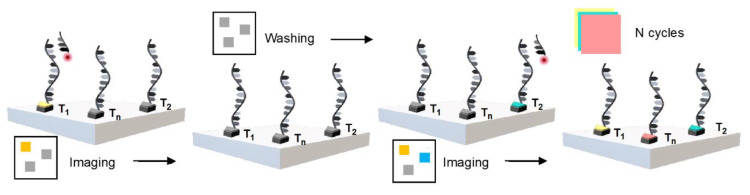
Presentative illustration of Exchange-PAINT.

It is difficult to obtain a high-resolution image of interest by PAINT, owing to the interaction between the dyes and the sample via hydrophobic or electrostatic coupling; this makes it difficult to simultaneously obtain images of a specific selection. In 2014, Jungmann et al. first introduced Exchange-PAINT, which was a multiplex image obtained using a single dye with one laser source targeting several spots [44]. They applied a pseudocolor to 10 images of origamis and four-color imaging to the cellular structure using a streptavidin-binding-based antibody to target special proteins for simultaneous imaging. Exchange-PAINT is a simple method for developing multiplexed imaging in one sample with DNA-PAINT (Figure 10). Typically, an elevated technique involves repeated steps of washing and diluting with imagers that bind with different dye-labeled docking sites. Thus, the proteins that conjugate with the docking strands can easily be labeled using imager strands, which are specific to their target proteins [45].

Lately, the Exchange-PAINT has been converted into Exchange-STORM and Exchange-STED, which has improved the multiplexed imaging over the conventional sequence-based labeling and constant labeling through stable hybridization, with an imager strand existing perpetually [46]. Agasti et al. developed a versatile labeling platform for the conjugation of DNA oligonucleotides to various labeling probes for DNA-PAINT and Exchange-PAINT, with a high labeling density, spatial accuracy, and achievable resolution [47]. Dai et al. examined a 5 nm gap between the docking sites on DNA origami and demonstrated, using combined multiplexed Exchange-PAINT imaging, a 5 × 5 nm pixel-sized nanodisplay with a <1 nm cross-channel registration accuracy [48]. Furthermore, Werbin et al. used Exchange-PAINT to determine the modification in a signal transduction, based on changes in the receptors of tyrosine kinases over time. In their study, it was concluded that the high-resolution imaging of complex receptors on a plasma membrane could reveal the different events of signal transduction based on each ligand [49].

**Figure 11:**
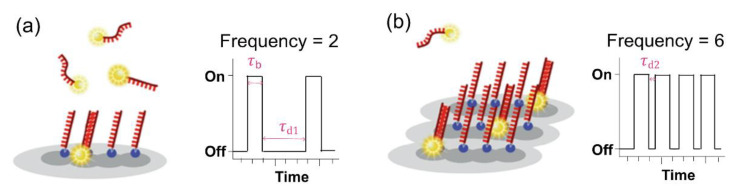
Principle of quantitative PAINT (qPAINT). (**a**) Intensity versus fluorescence on and off times (*τ*_b_ and *τ*_d_, respectively), characterized by the transient binding of fluorescence-labeled imager strands and docking strands. (**b**) High frequency of the imager strand binding with docking sites, with time trace versus intensity. The images were reproduced with permission from Lin et al., published by Academic Press Inc. Elsevier Science, 2020 [51].

To facilitate the counting of many molecules in the entire space, the development of quantitative PAINT (qPAINT) is required. Jungmann et al. established a method to quantify the DNA-PAINT analysis [50]. The uniformity of DNA-binding and dissociation without bleaching enables the elucidation of a quantitative image, which is applied in qPAINT. qPAINT is based on the analysis of the kinetic binding frequency between imager and docking strands, which are count targets. In the broad dynamic range, an alterable influx frequency of the imager strand enables high validity. This process can be explained with a second-order association rate (*k*_on_) and a first-order dissociation rate (*k*_off_) (Figure 11). The determination of “on” (*τ*_b_ is bright time) and “off” (*τ*_d_ is dark time) is associated with *k*_off_ and *k*_on_, respectively, by the following equations: (a) *τ*_b_ = *k*_off_^−1^; (b) *τ*_d_ = (*k*_on_ × *c*_i_)^−1^ = ξ^−1^, where ξ represents the influx rate of the imager strand and *c*_i_ represents the concentration of the imager strands.

**Figure 12:**
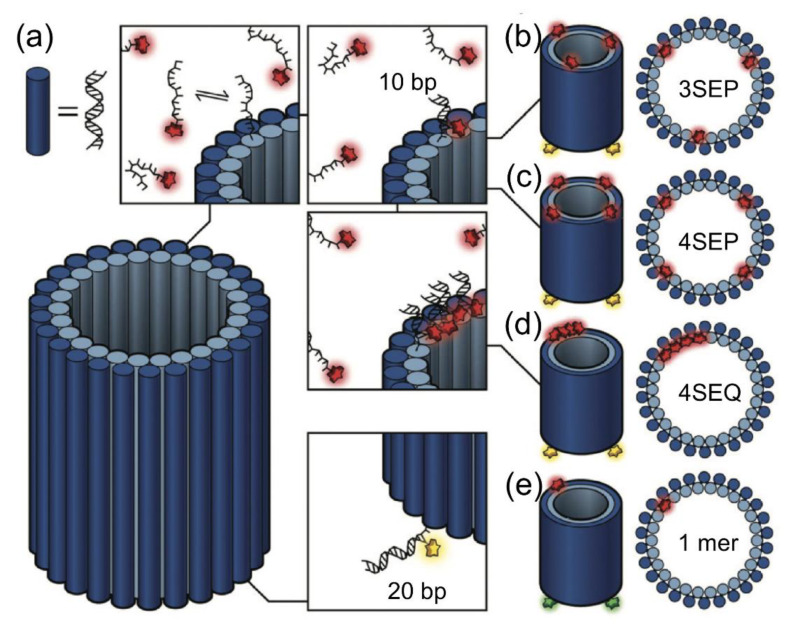
DNA nanotube. (**a**) 52 helix nanotube with 3′-DNA staples integrated with the DNA-PAINT docking strand (10 nt), complementary to the Alexa647-labeled imager strand. Multimeric nanotubes with (**b**) 3SEP, which have three docking sites at the top; (**c**) 4SEP, which integrates four PAINT docking sites at the top with a gap of approximately 15 nm; and (**d**) 4SEQ, which has four close docking sites separated by approximately 3 nm. Each nanotube base has four 20 nt staples, which are complementary to the oligonucleotide labeled with ATTO565 (for 3SEP, 4SEP, and 4SEQ) and Alexa488 (for 1mer). (**e**) For qPAINT calibration, a 1mer single-site nanotube is used. The images were reproduced with permission from Baker et al., published by the Royal Society of Chemistry, 2019 [53].

DNA-PAINT provides quantitative insights into the distribution of the available functional sites at both interparticle and intraparticle levels, offering additional information for the rational design of these nanomaterials. DNA-PAINT could be used to image and quantify relevant functional proteins, such as antibodies and streptavidin, on nanoparticles and microparticles, with nanometric accuracy in three dimensions and multiple colors [52]. Moreover, qPAINT is capable of imaging the small distance site of a DNA nanotube, which would allow access to the DNA helix structure. The docking strand needs to be in a close parallel position, a gap of approximately 3 nm (Figure 12) [53].

## 3. Conclusions

Numerous SMLM methods have been developed to localize single molecules by overcoming diffraction limitations; these methods play an important role in the determination of biological and/or physical significance. Furthermore, DNA nanoarchitectures can be applied as a scaffold for the assembly of nanodevices and 3D SR imaging, based on their natural diverse structure and hybridization in self-assembly. Therefore, the combination of DNA nanostructures (e.g., DNA origami) and SR technology offers both shape customization at the nanometer range and high-accuracy molecular-scale positioning and patterning.

Currently, SMLM still exhibits a lower imaging speed and resolution in real-time single-cell detections compared with those of SR imaging techniques. However, it affords details of a single-molecule structure in subcellular localization. The improvement of SMLM can be directed toward optical control, e.g., high-speed camera performance with other uses or different detection methods and chemical control, e.g., buffer solutions, dyes, and proteins. The camera is a major factor for achieving a high frame rate in SMLM. For example, the combination of a scientific CMOS (sCMOS) camera and SMLM provide a field of view of ~300 × 300 μm^2^ and a frame rate of up to 400 fps [54]. Moreover, fluorescence emitters must be improved to obtain sufficient photon output to match the imaging speed. Previously, Xu et al. applied dual-objective STORM in 3D imaging to enhance image quality by increasing the output of photons in each acquisition [55]. In chemical control, a specific protein (i.e., Agos [56]) loading is employed to increase the binding speed of the imager stand to the target site as well as increase the imaging speed up to 30 times using the combination of FRET and DNA-PAINT [43]. Moreover, the combination of a detection method along with various labeling dyes allows the monitoring of the interaction between dye and DNA molecule, which allows precise structural localization in customizable applications [57]. Thus, in SMLM using DNA nanostructures, it is important to expand the improvement of dynamic investigation in the single-molecule level through the elucidation of the structural behavior of DNA assembly.

As discussed above, stochastic switching-based localization microscopy is not only limited by the photon number per “on” time but also by the photoactivated fluorophore number, which can provide adequate signals that can be localized proximately in one diffraction-limited space. Thus, fluorescent probes with high photostability and additional functionalities, such as switching, are important targets for high-resolution imaging techniques. In addition, the development of suitable standards and probes for SR imaging in the near future will improve the accessibility of this tool for biologists and material scientists over the next few years, providing new insights into nanoscale phenomena. New methods for integrating biomolecules into DNA nanoarchitectures are becoming increasingly complex, and the application of DNA nanostructures in combination with SR technology applications offers great potential in biosensing for future studies in the fields of structural biology and biochemistry.
